# Astronauts as a Human Aging Model: Epigenetic Age Responses to Space Exposure

**DOI:** 10.1111/acel.70360

**Published:** 2026-01-11

**Authors:** Matías Fuentealba, JangKeun Kim, Jeremy Wain Hirschberg, Bader Shirah, Eliah G. Overbey, Christopher Mason, David Furman

**Affiliations:** ^1^ Buck AI Platform, Buck Institute for Research on Aging Novato California USA; ^2^ Department of Physiology and Biophysics Weill Cornell Medicine New York New York USA; ^3^ Department of Neuroscience King Faisal Specialist Hospital & Research Centre Jeddah Saudi Arabia; ^4^ Center for STEM University of Austin Austin Texas USA; ^5^ Stanford 1000 Immunomes Project Stanford School of Medicine Stanford California USA

## Abstract

Spaceflight exposes astronauts to a combination of environmental stressors such as microgravity, ionizing radiation, circadian disruption, and social isolation that induce phenotypes of aging. However, whether these exposures accelerate biological aging remains unclear. In this exploratory study, we assessed 32 DNA methylation‐based biological age metrics in 4 astronauts during the Axiom‐2 mission at pre‐flight, in‐flight (day 4 and 7), and post‐flight (return days 1 and 7). On average, Epigenetic Age Acceleration increased 1.91 years by flight day 7. Upon return to Earth, biological age decreased in all crew members, with older astronauts returning to pre‐flight estimates and younger astronauts showing a biological age significantly lower than pre‐flight levels. We found that shifts in immune cell composition, specifically regulatory and naïve CD4 T‐cells, accounted for a significant portion of the observed age acceleration in several clock models. However, even after adjusting for cell composition, chronological age and mortality‐based predictors showed acceleration during spaceflight. These findings suggest that spaceflight induces rapid, yet reversible, epigenetic changes associated with aging, positioning spaceflight as a platform to study human aging mechanisms and test geroprotective interventions.

## Graphical abstract

This longitudinal analysis of Axiom‐2 astronauts reveals that spaceflight induces a rapid andsignificant epigenetic age acceleration that is largely reversible upon return to Earth. These findings identify space exposure as a unique model for studying aging mechanisms and potentially testing geroprotective interventions.

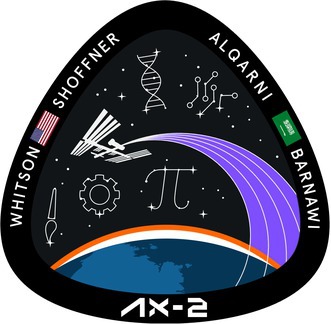

Aging can be conceptualized as a progressive process in which environmental stressors overwhelm the body's compensatory mechanisms, resulting in the accumulation of cellular damage that ultimately leads to functional decline and age‐related diseases (Franceschi et al. 2000; Polsky et al. 2022; Bektas et al. 2018; Lavretsky and Newhouse 2012). Space exposure constitutes a unique and intense combination of environmental stressors, including microgravity, ionizing radiation, circadian disruption, and social isolation, all of which elicit hallmark features of aging on Earth. For instance, microgravity‐induced mechanical unloading accelerates muscle atrophy and bone demineralization, mimicking degenerative processes typically observed in aging populations (Vico et al. 2000). Similarly, ionizing radiation induces DNA damage, promoting cellular senescence, and increasing susceptibility to cardiovascular disease and cancer (Garrett‐Bakelman et al. 2019). In addition, multi‐omics and cellular analyses from astronauts on various missions have revealed striking similarities between changes induced by spaceflight and those observed as we age, including alterations in the activation of mitochondrial stress response pathways (da Silveira et al. 2020), production of pro‐ and anti‐inflammatory cytokines (Capri et al. 2023), and expression of genes related to immune function and DNA repair (Garrett‐Bakelman et al. 2019).

In recent years, researchers have developed dozens of biological aging clocks, which estimate cumulative physiological decline and potentially gauge how many healthy years remain based on DNA methylation data (Hannum et al. 2013; Horvath 2013; Levine et al. 2018; Belsky et al. 2022; Fuentealba et al. 2025). Despite the remarkable similarity between the effects of space exposure and aging on Earth (Campisi et al. 2024), whether space exposure influences biological age in humans remains unclear. A previous study in mice flown for 37 days to the International Space Station (ISS) revealed that spaceflight decelerated the DNA methylation age of the retina compared to ground‐based controls (Chen et al. 2021). Similarly, a study of 6 participants in the Mars‐500, a 520‐day psychosocial isolation experiment, found that social isolation was associated with a decrease in epigenetic aging relative to baseline (Nwanaji‐Enwerem et al. 2020). Also, a 1‐year mission to the ISS conducted by astronaut Scott Kelly showed an increase in telomere length in white blood cells (Garrett‐Bakelman et al. 2019).

In this study, we collected blood samples from astronauts aboard the Axiom‐2 mission, a commercial 9‐day mission to the ISS. The mission included four astronauts: A1 (male, 67.7 years), A2 (female, 63.2 years), A3 (male, 31.1 years), and A4 (female, 34.6 years). We collected blood samples at five timepoints: 45 days before liftoff (L‐45), during spaceflight on days 4 (FD + 4) and 7 (FD + 7), and after return on days 1 (R + 1) and 7 (R + 7). We profiled DNA methylation using the Illumina Methylation EPIC array and computed 32 epigenetic clocks (Table S1). Among the 31 epigenetic clocks expressed in units of years (i.e., excluding DunedinPACE), Pearson's correlation coefficient with chronological age ranged from 0.66 (AdaptAge) to 0.99 (PCHorvath1) with 28 of the 31 clocks showing a correlation above 0.9 (Figure S1).

We calculated the effects on biological age using three approaches: (1) “Epigenetic Age Difference” (EAD) corresponding to the difference between epigenetic age and chronological age, (2) “Epigenetic Age Acceleration” (EAA) indicating the epigenetic age adjusted by chronological age and sex, and (3) “Intrinsic Epigenetic Age Acceleration” (IEAA) as the epigenetic age adjusted by age, sex and predicted cell composition of 12 cell types (Table S2). Whereas EAA reflects deviations from the expected epigenetic age for a given chronological age and sex, including influences of cell composition changes, IEAA aims to reflect only age‐associated epigenetic changes within the cells rather than cell type shifts. In the case of EAA, correlations within the same individual across timepoints were higher than between the same timepoints in different individuals (Figure S2). However, IEAA showed lower correlations within the same individual than within the same timepoint.

On average, astronaut A1 exhibited the greatest increase in EAD and EAA at FD + 4 with 2.43 and 2.45 years compared to L‐45, followed by A2 with 1.18 and 1.2 years and A4 with 1.04 and 1.06 years, respectively (Figure 1a, Table S3). In contrast, crew member A3 showed a significant decrease of −1.66 years in EAD (*p*_perm_ < 1.00e‐04) and −1.64 years in EAA (*p*_perm_ < 1.00e‐04). On average, astronauts increased 0.74 years EAD (*p*_LMM_ = 0.31) and 0.76 years EAA (*p*_LMM_ = 0.10) after 4 days in space. By FD + 7, astronauts increased EAD and EAA by 1.14 years on average compared to FD + 4 (EAD *p*_LMM_ = 0.03, EAA *p*_LMM_ = 1.97e‐04), and by 1.89 years (EAD *p*_LMM_ = 0.01) and 1.91 years (EAA *p*_LMM_ = 1.09e‐04) compared to L‐45.

**FIGURE 1:**
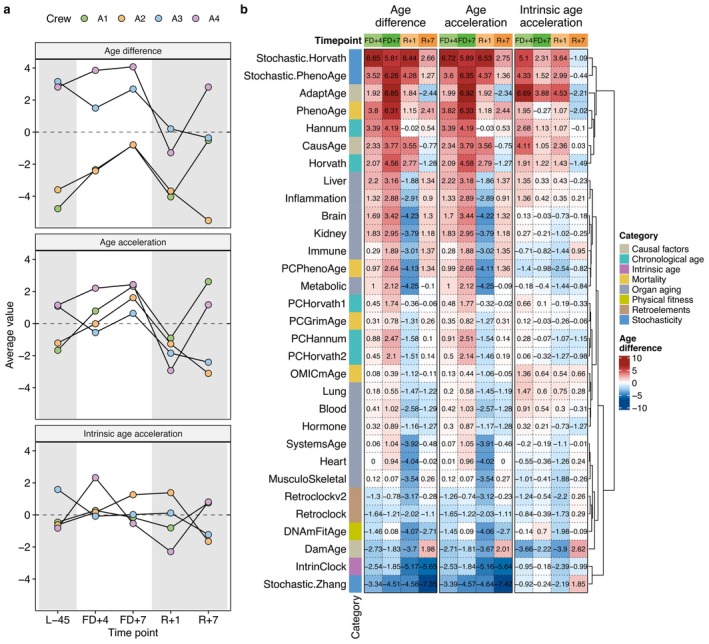
Dynamics of biological age during and after spaceflight. (a) Longitudinal trajectories of epigenetic age estimates in astronauts (A1, A2, A3, A4) across timepoints: Pre‐flight (L‐45), in‐flight (FD+4, FD+7), and return (R+1, R+7). The panels display three distinct metrics: Epigenetic Age Difference (EAD, unadjusted), Epigenetic Age Acceleration (EAA, adjusted for age and sex), and Intrinsic Epigenetic Age Acceleration (IEAA, adjusted for age, sex, and cell composition). Data points represent the mean value across 31 epigenetic clocks for each astronaut. Background shading indicates the mission phase (white = in‐flight). (b) Heatmap displaying the change in biological age (in years) for each specific clock relative to pre‐flight (L‐45) baseline levels. Rows correspond to 31 epigenetic clocks grouped by functional category (e.g., Chronological age, Mortality, and Organ aging) as indicated by the colored annotation bar. Columns are stratified by the calculation method (EAD, EAA, and IEAA) and timepoint. Cell color intensity represents the magnitude of the biological age shift, with red indicating acceleration and blue indicating deceleration compared to baseline.

We also determined biological age 1 day after return to Earth (R + 1) and found that all astronauts showed a decrease in EAD and EAA compared to FD + 7, with an average reduction of −3.49 (EAD p_LMM_ = 1.66e‐08) and −3.48 (EAA p_LMM_ = 7.6e‐15) years. In fact, EAA in A1 and A2 (older astronauts) returned to levels not significantly different from L‐45 (*p*_perm_ = 0.70 and 0.87, respectively). In contrast, younger crew members A3 (*p*_perm_ = 3.50e‐03) and A4 (*p*_perm_ = 4.00e‐04) exhibited biological ages significantly below pre‐flight levels (−2.93 and −4.08 years, respectively). However, this reduction was transient for A4 who returned to baseline by R + 7 (*p*_perm_ = 0.95) but persisted in A3. These results suggest that older astronauts may exhibit more pronounced epigenetic age fluctuations during spaceflight, supporting the idea that aging impinges on cellular resilience, stress‐response plasticity (Franceschi et al. 2018) and promotes instability of regulatory networks (Horvath and Raj 2018).

We also analyzed DunedinPACE, which is the only epigenetic clock that estimates the rate of aging instead of biological age, and observed that it tended to increase at FD + 4 compared to L‐45 (mean increase compared to L‐45 = 0.01), then declined in all astronauts at FD + 7 (mean decline compared to FD + 4 = −0.03), and continued to decline at R + 1 (mean decline compared to FD + 4 = −0.08) (Figure S3). Interestingly, astronauts A1, A3, and A4 displayed a lower rate of aging after return compared to pre‐flight (−0.13, −0.07, and −0.10, respectively).

We adjusted the biological age by cell composition (i.e., 12 cell types) estimates from DNA methylation in addition to sex and age. Compared to L‐45, astronauts A2 and A4 showed a statistically significant increase of 0.79 years (*p*_perm_ = 4.20e‐02) and 3.15 years (*p*_perm_ < 1.00e‐04) at FD + 4, which only remained in A2 at FD + 7 with 1.88 years (p_perm_ < 1.00e‐04). In addition, A3 and A4 (younger astronauts) also showed lower IEAA at R + 1 (−1.47 years in both cases), compared to L‐45, while A2 displayed accelerated IEAA by 2.01 years at R + 1 (*p*_perm_ < 1.00e‐04) and A1 showed no change (*p*_perm_ = 0.69). On average, astronauts exhibited an increase of 0.75 years in estimated biological age after 4 days in space (*p*_LMM_ = 8.56e‐04), but only 0.22 after 7 days (*p*_LMM_ = 0.18), and then decreased −0.32 years (*p*_LMM_ = 0.22) at R + 1 and −0.24 years (p_LMM_ = 0.30) at R + 7 compared to pre‐flight (L‐45).

These results indicate that the increase in EAA observed during spaceflight (particularly FD + 7) and the subsequent deceleration after return (R + 1) were, for many clocks, mediated by cell composition changes rather than aging‐associated epigenetic shifts which only remained at FD + 4. However, these adjusted estimates should be interpreted with caution given the cell proportions are predicted values and calculated from the same DNA methylation data used to estimate the biological aging clocks. Moreover, collinearity between CpG sites used in the deconvolution and the epigenetic clocks will result in adjustments of the biological age estimates toward zero. Thus, accurate cell composition adjustments require experimental measurements alongside DNA methylation profiling.

We asked which cell types mediated this difference by applying a dominance analysis to a model in which the outcome was the difference between EAA and IEAA to determine the contribution of each of the 12 cell types. We found that memory regulatory T‐cells (*R* ^2^ = 0.20) explained 20% of the variation in how EAA changes relative to IEAA, followed by naïve CD4 T cells (*R* ^2^ = 0.16), and neutrophils (*R* ^2^ = 0.10) (Table S4). The predicted proportion of regulatory T‐cells and naïve CD4 T‐cells tended to decrease at FD + 4 and then increase at FD + 7, to decrease again after return to Earth (Figure S4). Neutrophils showed the opposite trend, with a decrease in proportion at FD + 7 and then a return to pre‐flight levels. Importantly, the deconvolution reference matrix used (Luo et al. 2023) shows slightly higher prediction errors for T‐regulatory compared to the original deconvolution library (T‐regulatory RMSE 2.6% vs. 1.34%) (Salas et al. 2022), which may add noise to age acceleration adjustments.

Although we considered only epigenetic clocks expressed in units of years when averaging EAD, EAA, and IEAA, each clock is trained on a different outcome sometimes using a restricted set of features in the process. Thus, some clocks may be more sensitive to changes induced by spaceflight, whereas others may be more sensitive to changes in blood cell composition. To investigate this, we grouped the 31 epigenetic clocks into nine categories according to their predicted outcomes and features used: chronological age (Horvath, Hannum, PCHorvath1, PCHorvath2, PCHannum), mortality (PhenoAge, OMICmAge, PCPhenoAge, PCGrimAge), physical fitness (DNAmFitAge), causal factors (AdaptAge, CausAge, DamAge), intrinsic age (IntrinClock), stochasticity (Stochastic.Zhang, Stochastic.Horvath, Stochastic.PhenoAge), retroelements (Retroclock, Retroclockv2), organ aging (SystemsAge, Blood, Brain, Inflammation, Heart, Hormone, Immune, Kidney, Liver, Metabolic, Lung, and MusculoSkeletal) (Figure 1b).

We first evaluated the overall correlation between the categories of clocks. When comparing EAA, clocks built on fitness parameters, retroelements, and intrinsic age (IntrinClock) were correlated. Also, organ aging clocks and mortality‐based clocks were correlated. Chronological age predictors showed a moderate and similar correlation with all other clocks (Figure S5). When considering IEAA, similar clusters were observed; however, the correlations between the clocks were on average higher.

The average EAA trends revealed that stochastic clocks showed the greatest increase at FD + 4 compared to L‐45 (2.30 years), followed by chronological age (1.47 years) and mortality‐based predictors (1.32 years) (Figure S6, Table S5). In contrast, the cluster of clocks built on physical fitness, retroelements, and intrinsic age displayed negative EAA (−1.44, −1.45, and −2.53 years, respectively). At FD + 7, a similar trend was observed; however, chronological age predictors showed the greatest acceleration with 3.03 years, followed by causal clocks (2.96 years) and mortality‐based predictors (2.56 years). Only the IntrinClock and clocks built on retroelement factors displayed a decrease in biological age at FD + 7 compared to L‐45 (−1.83 and −0.97 years, respectively). Except for stochastic and causal clocks, all categories showed a decrease in EAA at R + 1 compared to pre‐flight (L‐45). Importantly, when we calculated IEAA, we observed the same effects as in EAA, with chronological age predictors, stochastic and causal clocks showing a positive acceleration at FD + 4 or FD + 7 compared to L‐45, and clocks built on retroelements and intrinsic age showing a negative acceleration.

On average across epigenetic clock categories, IEAA increased by 0.62 at FD + 4 and 0.29 at FD + 7 compared to L‐45 and resulted in −0.60 years 1 day after return and −0.25 years 7 days after return. This result indicates that epigenetic clocks are differentially affected by cell composition changes and that spaceflight exposure is associated with an acceleration of biological age even after cell composition adjustment, particularly in chronological age and mortality‐based clocks. Overall, the biological age effects occurred during early flight, followed by a compensatory decrease by FD + 7. This downward trajectory continued upon return, reaching biological ages below pre‐flight, before rebounding toward stabilization 1 week after return (R + 7).

The sample size of four astronauts limits how far we can generalize these findings. Although we used linear‐mixed models (for estimating significance across astronauts) and a permutation‐based approach (for estimating significance between timepoints) that considers the dependence between the clocks and repeated measure design, resulting in conservative estimates, we should treat the *p* values and effect sizes as exploratory and confirm them in larger cohorts and longer missions. Another major limitation is the lack of an Earth‐based control group. Diet shifts toward freeze‐dried foods, altered sleep schedules (circadian rhythm alterations), and reduced activity all influence DNA methylation and blood cell composition, creating confounding effects that cannot be removed with within‐participant comparisons. Also, recent studies have shown that epigenetic age oscillates during the day and variation in collection time could contribute to the observed noise (Koncevičius et al. 2024). Future studies need parallel ground crews to isolate the specific biological effects of spaceflight. The modest but rapid fluctuations in biological age observed during and after spaceflight documented here highlight the potential of space missions as a platform to test interventions that may slow aging and functional decline. Thus, spaceflight countermeasures could serve as candidate antiaging therapies on Earth, while geroprotective interventions could be repurposed to support astronaut health. Future longitudinal studies on longer missions will be critical to determine the persistence of biological age changes and assess the effectiveness of countermeasures such as exercise, nutritional supplementation, and other interventions.

## 1 Methods

### 1.1 Axiom‐2 Mission

Axiom Mission 2 (Ax‐2) was a 9‐day, 5‐h, 26‐min private astronaut flight to the International Space Station (ISS) operated by Axiom Space in partnership with NASA and SpaceX. The mission launched aboard SpaceX's Crew Dragon *Freedom* on a Falcon 9 from Launch Complex 39A, Kennedy Space Center, at 21:37 UTC on 21 May 2023, marking the second all‐commercial crewed visit to the ISS. *Freedom* docked to the Harmony zenith port on 22 May and remained attached for 8 days. Biospecimens were collected under standard ISS protocols during these studies form the basis of the analyses reported here. The spacecraft undocked on 30 May and splashed down in the Gulf of Mexico off Panama City, Florida, in the early hours of 31 May 2023, successfully concluding Axiom Space's second pathfinder mission.

### 1.2 Blood Sample Collection

We collected venous blood from the 4 astronauts of the Axiom Mission 2 before, during, and after a short‐duration mission onboard the ISS. Baseline (preflight) sampling occurred 45 days before launch (Launch (L) L‐45). In‐flight sampling occurred on (Flight Day (FD) +4 and +7 days). Postflight sampling occurred on (Return (R) R+1, R+7). Trained and certified astronauts performed in‐flight blood draws using standard phlebotomy techniques (venipuncture). We collected non‐fasted venous blood into plasma preparation tubes (PPTs) (BD Biosciences, cat # 362788). At R+1 only, we used K2 EDTA tubes in substitution of PPTs. The amount of blood drawn was 5 mL per astronaut at each timepoint. After collection, we centrifuged whole blood at room temperature at 1256 g for 25 min and then frozen at −80°C within 60 min of sampling. Then, we shipped the samples to Weill Cornell Medicine. The remaining samples now reside in the Cornell Aerospace Biobank (CAMbank) and all metadata is securely stored at the HRH Prince Alwaleed Bin Talal Bin Abdulaziz Alsaud Institute for Computational Biomedicine (Weill Cornell Medicine, New York, United States).

### 1.3 DNA Extraction

We extracted high molecular weight (HMW) genomic DNA (gDNA) from the cell pellet below the gel barrier of the BD Vacutainer PPT tube (cat # 362788) following centrifugation, using the Monarch HMW DNA Extraction Kit for Cells & Blood (cat # T3050L). We sheared the extracted HMW gDNA using Covaris G‐Tubes (cat # 520079) down to a target size of 20 kb. We measured fragment length using Genomic ScreenTape (Agilent, cat # 5067 5365) on the Agilent 4200 TapeStation System (cat # G2991BA). We quantified DNA using the Qubit 1X dsDNA High Sensitivity Assay Kit (cat # Q33230) on the Qubit 4 Fluorometer (cat # Q33238). We measured the quantity and fragment length before and after shearing.

### 1.4 DNA Methylation Determination and Processing

We assessed DNA methylation using the TruAge epigenetic age platform (TruDiagnostic Inc., Lexington, KY). We collected and lysed peripheral whole blood in a stabilization buffer. We extracted genomic DNA and bisulfite converted 500 ng using the EZ DNA Methylation Kit (Zymo Research) following the manufacturer's instructions. We hybridized the converted DNA to the Illumina Infinium MethylationEPIC v2.0 BeadChip (850 k) with the custom aging add‐on available from TruDiagnostic. We randomly assigned samples to BeadChip wells to reduce technical batch effects. We scanned arrays using the Illumina iScan SQ platform, generating IDAT files for each sample. We processed raw intensity data using the minfi R package (Aryee et al. 2014). We performed quality control using ENmix (Xu et al. 2021), identifying outliers based on internal control probe variance (> 3 SD from the mean); no outliers were detected. We applied single‐sample Noob (ssNoob) normalization to correct for background and dye bias. We calculated detection *p* values and removed all samples with a mean detection *p* value > 0.05. Additionally, we excluded CpGs failing detection in more than 5% of samples (*p* > 0.05). After QC, we collapsed probes unique to EPIC v2 arrays and imputed missing probes (relative to EPIC v1) using blood‐specific medians from the sesameData package (Zhou et al. 2025 ). The final normalized beta matrix was used for downstream analysis.

### 1.5 Biological Age Calculation

We calculated epigenetic age difference (EAD) as the difference between epigenetic age and chronological age. We quantified epigenetic age acceleration (EAA) as the residual deviation of each DNA methylation‐based age estimate from its expected value given chronological age and sex. For every clock—including principal component–based clocks (PCHorvath1, PCHorvath2, PCHannum, PCPhenoAge, and PCGrimAge) (Higgins‐Chen et al. 2022), first‐generation clocks (Horvath (Horvath 2013), Hannum (Hannum et al. 2013)), PhenoAge (Levine et al. 2018), OMICmAge (Chen et al. 2023), DNAmFitAge (McGreevy et al. 2023), causal clocks (AdaptAge, CausAge, and DamAge) (Ying et al. 2024), the intrinsic clock (IntrinClock) (Tomusiak et al. 2024), stochastic clocks (Stochastic.Zhang, Stochastic.Horvath, and Stochastic.PhenoAge) (Tong et al. 2024), retroelement‐based clocks (Retroclock and Retroclockv2) (Ndhlovu et al. 2024), and 12 organ‐system clocks (Blood, Brain, Inflammation, Heart, Hormone, Immune, Kidney, Liver, Metabolic, Lung, MusculoSkeletal, and SystemsAge) (Sehgal et al. 2025). We fitted a separate linear regression model of the form:

$$\begin{array}{c} \text{Epigenetic}\;\mathrm{Age}=\beta ₀+\beta ₁\cdot \left(\text{Chronological}\;\mathrm{Age}\_i\right)\\ \;+\beta ₂\cdot \left(\mathrm{Sex}\_i\right)+\varepsilon \_i \end{array}$$

where Sex was encoded as Female = 1 and Male = 0. The residual ε_i, extracted via R's resid() function, represents the EAA for sample i, (i.e., the residual epigenetic age after adjusting for age and sex). We predicted cell composition using a DNA methylation reference matrix (Luo et al. 2023), which deconvolutes 12 immune cell subtypes based on Illumina 850 k DNAm profiles of cell‐sorted samples from Salas et al. (2022). We calculated intrinsic epigenetic age acceleration (IEAA) by fitting a linear model of epigenetic age on chronological age, sex, and the predicted fraction of the 12 cell types, and extracting the residual (ε_i).

### 1.6 Significance Testing

Given the limited number of samples, we evaluated the changes in biological age (EAD, EAA, and IEAA) for each crew member across timepoints. We treated the set of 31 epigenetic clocks expressed in units of years as repeated measures. We employed two statistical approaches to compare timepoints within each subject: (1) We initially assessed differences using a two‐sided paired Wilcoxon signed‐rank test. This nonparametric test compares the paired distributions of biological estimates across clocks to determine if the median difference between timepoints differs from zero. (2) To account for the high correlation between epigenetic clocks, which violates the independence assumption of standard tests, we implemented a permutation‐based test on the mean differences (i.e., *p*_perm_). For each astronaut and timepoint, we calculated the vector of paired differences for all clocks. We then constructed a null distribution by randomly assigning a positive or negative sign to each difference and calculating the mean of these sign‐flipped values in 10,000 permutations. Finally, we calculated the two‐sided *p* values as the proportion of permuted means with an absolute value greater or equal to the absolute value of the observed mean difference.

In addition, we calculated overall statistically significant changes between timepoints across astronauts and epigenetic clocks using linear mixed models (i.e., *p*_LMM_). We included timepoint as a fixed effect while considering random intercepts for both the crew member and the epigenetic clock to account for repeated measures and between‐clock variability.

$$\text{Epigenetic}\;\mathrm{Age}=\beta ₀+\beta ₁\cdot \left(\text{Timepoint}\_k\right)+b\_\text{0i}+c\_\text{0j}+\varepsilon \_\mathrm{ijk}$$

where b_0i represents the random intercept for Astronaut i (accounts for 1|crew) and c_0j represents the random intercept for Clock j (accounts for 1|clock). We calculated this using the function lmer from the lme4 R package.

Considering that the use of different epigenetic clocks as repeated measures might be sensitive to the influence of specific clocks with large variation, we performed a sensitivity analysis where we removed one clock and a time and repeated the calculations for mean, Wilcoxon test and permutation‐based test. Overall, when *p* values were below 0.05 in the test with all the clocks included, they remained below 0.05 when an individual clock was excluded (Figure S7). The only exception was the removal of AdaptAge in A1 for the age difference using a permutation‐based approach which resulted in a *p* = 5.04e‐2.

## Author Contributions

M.F., D.F., and C.M. conceptualized the project. C.M. and D.F. provided supervision. M.F. performed the formal data analysis and generated the visualizations. J.K., J.W.H., B.S., and E.G.O. coordinated the collection of astronaut samples and oversaw their shipment to TruDiagnostic Inc. for DNA methylation profiling. M.F. and D.F. drafted the original manuscript, and all authors reviewed and approved the final version.

## Funding

The authors have nothing to report.

## Conflicts of Interest

D.F. is co‐founder of Cosmica Biosciences. All other authors have declared no competing interests.

## Supporting information


**Table S1:** List of epigenetic clocks used in this study, with the corresponding original reference publication.
**Table S2:** List of the 12 DNA methylation derived immune cell types used for cell composition adjustment.
**Table S3:** Biological age differences for each crew member across different timepoints. For each biological age measure (EAD, EAA, and IEAA), the table describes the mean difference (Time 2—Time 1), standard error (SE), 95% confidence interval, and *p* values from paired Wilcoxon signed‐rank tests and sign‐flipping permutation tests on the paired differences in biological age. All *p* values reported in the main text correspond to the permutation‐based *p* values.
**Table S4:** Variance explained DNA methylation‐estimated immune cell types in the difference between epigenetic age acceleration and epigenetic intrinsic age acceleration.
**Table S5:** Mean change in age acceleration and intrinsic age acceleration between mission timepoints (Time 2—Time 1) for each epigenetic clock category.
**Figure S1:** Distribution of epigenetic age estimates for each clock. Clocks are ordered by their correlation with chronological age (red labels).
**Figure S2:** Correlation between the biological age changes across astronauts and timepoints. Top panels display the pairwise Pearson's correlations for EAD, EAA, and IEAA. Bottom panels display the distribution of correlations stratified by comparison type (different individuals (any timepoint), different timepoints (any individual), same individual (any timepoint), and same timepoint (any individual)) for each measure (EAD, EAA, and IEAA).
**Figure S3:** Changes in the pace of aging for each astronaut across timepoints. Points indicate individual DunedinPACE values by crew member.
**Figure S4:** DNA methylation estimated immune cell type proportions across timepoints. Each panel shows the predicted proportions for one of the 12 cell types.
**Figure S5:** Pairwise Pearson's correlation between categories of epigenetic clocks for the biological age measures.
**Figure S6:** Average biological age change for different epigenetic clock categories.
**Figure S7:** Sensitivity analysis of biological age changes across epigenetic clocks using a leave‐one‐out approach.

## Data Availability

The data that support the findings of this study are openly available in github at https://github.com/msfuentealba/aging_space.
