# Supplementary material for: Astronauts as a Human Aging Model: Epigenetic Age Responses to Space Exposure
Source: Aging Cell. 2026 Jan 11;25(2):e70360. doi: 10.1111/acel.70360 (PMC12791563; doi:10.1111/acel.70360)

Table S1. List of epigenetic clocks used in this study, with the corresponding original reference publication.

| **Epigenetic clock** | **Description** | **Source** |
| --- | --- | --- |
| AdaptAge | Adaption Age (causal clock) | https://www.nature.com/articles/s43587-023-00557-0 |
| CausAge | Causal Age (causal clock) | https://www.nature.com/articles/s43587-023-00557-0 |
| DamAge | Damage Age (causal clock) | https://www.nature.com/articles/s43587-023-00557-0 |
| DNAmFitAge | DNAm Fit Age | https://pubmed.ncbi.nlm.nih.gov/36812475/ |
| DunedinPACE | DunedinPACE | https://elifesciences.org/articles/73420 |
| PCGrimAge | GrimAge estimation (principal component) | https://pmc.ncbi.nlm.nih.gov/articles/PMC6366976/ |
| Hannum | Hannum clock (non-PC) | https://www.sciencedirect.com/science/article/pii/S1097276512008933 |
| PCHannum | Hannum clock (principal component) | https://www.nature.com/articles/s43587-022-00248-2 |
| Horvath | Horvath multi-tissue (non-PC) | https://pubmed.ncbi.nlm.nih.gov/24138928/ |
| PCHorvath1 | Horvath multi-tissue clock (principal component version) | https://www.nature.com/articles/s43587-022-00248-2 |
| PCHorvath2 | Horvath skin + blood clock (principal component) | https://www.nature.com/articles/s43587-022-00248-2 |
| IntrinClock | Intrinsic clock | https://www.nature.com/articles/s42003-024-06609-4 |
| OMICmAge | OMICmAge clock | https://pmc.ncbi.nlm.nih.gov/articles/PMC10614756/ |
| PhenoAge | PhenoAge / Levine clock (non-PC) | https://pmc.ncbi.nlm.nih.gov/articles/PMC5940111/ |
| PCPhenoAge | PhenoAge / Levine clock (principal component) | https://www.nature.com/articles/s43587-022-00248-2 |
| Retroclock | Retroviral based epigenetic age clock (using retroviral CpGs) | https://pubmed.ncbi.nlm.nih.gov/39092674/ |
| Retroclockv2 | Retroviral based epigenetic age clock (using retroviral CpGs) version 2 | https://pubmed.ncbi.nlm.nih.gov/39092674/ |
| Stochastic.Horvath | Stochastic clock using Horvath CpGs | https://www.nature.com/articles/s43587-024-00600-8 |
| Stochastic.PhenoAge | Stochastic clock using PhenoAge | https://www.nature.com/articles/s43587-024-00600-8 |
| Stochastic.Zhang | Stochastic clock using Zhang CpGs | https://www.nature.com/articles/s43587-024-00600-8 |
| Blood | SystemsAge - Blood Age | https://www.nature.com/articles/s43587-025-00958-3 |
| Brain | SystemsAge - Brain Age | https://www.nature.com/articles/s43587-025-00958-3 |
| Heart | SystemsAge - Heart age | https://www.nature.com/articles/s43587-025-00958-3 |
| Hormone | SystemsAge - Hormone age | https://www.nature.com/articles/s43587-025-00958-3 |
| Immune | SystemsAge - Immune age | https://www.nature.com/articles/s43587-025-00958-3 |
| Inflammation | SystemsAge - Inflammation system age | https://www.nature.com/articles/s43587-025-00958-3 |
| Kidney | SystemsAge - Kidney age | https://www.nature.com/articles/s43587-025-00958-3 |
| Liver | SystemsAge - Liver age | https://www.nature.com/articles/s43587-025-00958-3 |
| Lung | SystemsAge - Lung age | https://www.nature.com/articles/s43587-025-00958-3 |
| Metabolic | SystemsAge - Metabolic age | https://www.nature.com/articles/s43587-025-00958-3 |
| MusculoSkeletal | SystemsAge - MusculoSkeletal age | https://www.nature.com/articles/s43587-025-00958-3 |
| SystemsAge | SystemsAge - Total Systems age | https://www.nature.com/articles/s43587-025-00958-3 |

Table S2. List of the 12 DNA methylation derived immune cell types used for cell composition adjustment.

| **Cell type** | **Description** | **Source** |
| --- | --- | --- |
| Bmem | B memory percent based on DNAm | https://pmc.ncbi.nlm.nih.gov/articles/PMC10388560/ |
| Bnv | B naïve based on DNAm | https://pmc.ncbi.nlm.nih.gov/articles/PMC10388560/ |
| Baso | Basophil percent based on DNAm | https://pmc.ncbi.nlm.nih.gov/articles/PMC10388560/ |
| CD4Tmem | CD4T memory percent based on DNAm | https://pmc.ncbi.nlm.nih.gov/articles/PMC10388560/ |
| CD4Tnv | CD4T naive percent based on DNAm | https://pmc.ncbi.nlm.nih.gov/articles/PMC10388560/ |
| CD8Tmem | CD8T memory cells based on DNAm | https://pmc.ncbi.nlm.nih.gov/articles/PMC10388560/ |
| CD8Tnv | CD8T naïve memory cells based on DNAm | https://pmc.ncbi.nlm.nih.gov/articles/PMC10388560/ |
| Eos | Eosinophils based on DNAm | https://pmc.ncbi.nlm.nih.gov/articles/PMC10388560/ |
| Mono | Monocyte percent based on DNAm | https://pmc.ncbi.nlm.nih.gov/articles/PMC10388560/ |
| Neu | Neutrophil percent based on DNAm | https://pmc.ncbi.nlm.nih.gov/articles/PMC10388560/ |
| NK | NK cells based on DNAm | https://pmc.ncbi.nlm.nih.gov/articles/PMC10388560/ |
| Treg | Regulatory T cell based on DNAm | https://pmc.ncbi.nlm.nih.gov/articles/PMC10388560/ |

Table S3. Biological age differences for each crew member across different timepoints. For each biological age measure (EAD, EAA, IEAA), the table describes the mean difference (Time 2 - Time 1), standard error (SE), 95% confidence interval, and p-values from paired Wilcoxon signed-rank tests and sign-flipping permutation tests on the paired differences in biological age. All p-values reported in the main text correspond to the permutation-based p-values.

| **Crew** | **Time 1** | **Time 2** | **Age difference** | | | | | **Age acceleration** | | | | | **Intrinsic age acceleration** | | | | |
| --- | --- | --- | --- | --- | --- | --- | --- | --- | --- | --- | --- | --- | --- | --- | --- | --- | --- |
|  |  |  | **Mean** | **SE** | **CI** | **Wilcoxon p** | **Permutation p** | **Mean** | **SE** | **CI** | **Wilcoxon p** | **Permutation p** | **Mean** | **SE** | **CI** | **Wilcoxon p** | **Permutation p** |
| A1 | L-45 | FD+4 | 2.43 | 1.68 | [-1.00, 5.86] | 7.59e-02 | 1.64e-01 | 2.45 | 1.68 | [-0.99, 5.89] | 6.95e-02 | 1.63e-01 | 0.73 | 0.53 | [-0.35, 1.81] | 2.09e-01 | 1.81e-01 |
| A1 | L-45 | FD+7 | 3.97 | 1.7 | [0.50, 7.45] | 6.22e-03 | 2.44e-02 | 3.99 | 1.7 | [0.51, 7.47] | 5.82e-03 | 2.39e-02 | 0.29 | 0.33 | [-0.37, 0.96] | 5.29e-01 | 3.81e-01 |
| A1 | L-45 | R+1 | 0.74 | 1.91 | [-3.17, 4.64] | 6.92e-01 | 7.07e-01 | 0.76 | 1.92 | [-3.15, 4.67] | 6.78e-01 | 7.00e-01 | -0.35 | 0.87 | [-2.13, 1.43] | 3.67e-01 | 6.96e-01 |
| A1 | L-45 | R+7 | 4.26 | 1.7 | [0.79, 7.72] | 3.35e-03 | 1.70e-02 | 4.28 | 1.7 | [0.81, 7.75] | 3.35e-03 | 1.68e-02 | 1.21 | 0.4 | [0.38, 2.03] | 1.16e-03 | 4.60e-03 |
| A1 | FD+4 | FD+7 | 1.54 | 0.34 | [0.84, 2.24] | 2.22e-05 | 0.00e+00 | 1.54 | 0.34 | [0.85, 2.24] | 2.22e-05 | 0.00e+00 | -0.44 | 0.24 | [-0.94, 0.06] | 7.27e-02 | 8.66e-02 |
| A1 | FD+7 | R+1 | -3.24 | 0.54 | [-4.33, -2.14] | 2.99e-06 | 0.00e+00 | -3.23 | 0.54 | [-4.33, -2.14] | 2.99e-06 | 0.00e+00 | -0.65 | 0.59 | [-1.85, 0.55] | 6.35e-02 | 2.84e-01 |
| A1 | R+1 | R+7 | 3.52 | 0.67 | [2.15, 4.89] | 6.27e-05 | 0.00e+00 | 3.52 | 0.67 | [2.15, 4.89] | 6.27e-05 | 0.00e+00 | 1.56 | 0.89 | [-0.25, 3.37] | 1.98e-02 | 9.24e-02 |
| A2 | L-45 | FD+4 | 1.18 | 0.55 | [0.06, 2.30] | 6.22e-03 | 3.49e-02 | 1.2 | 0.55 | [0.09, 2.32] | 5.44e-03 | 3.18e-02 | 0.79 | 0.36 | [0.04, 1.53] | 4.56e-02 | 4.20e-02 |
| A2 | L-45 | FD+7 | 2.81 | 0.46 | [1.86, 3.75] | 7.01e-06 | 0.00e+00 | 2.83 | 0.46 | [1.88, 3.77] | 7.01e-06 | 0.00e+00 | 1.88 | 0.41 | [1.04, 2.72] | 2.22e-05 | 0.00e+00 |
| A2 | L-45 | R+1 | -0.07 | 0.32 | [-0.72, 0.57] | 9.92e-01 | 8.17e-01 | -0.05 | 0.31 | [-0.69, 0.59] | 1.00e+00 | 8.71e-01 | 2.01 | 0.4 | [1.19, 2.83] | 2.57e-06 | 0.00e+00 |
| A2 | L-45 | R+7 | -1.92 | 0.99 | [-3.95, 0.11] | 5.79e-02 | 5.16e-02 | -1.89 | 0.99 | [-3.92, 0.13] | 5.52e-02 | 5.31e-02 | -1.03 | 0.6 | [-2.25, 0.20] | 1.57e-01 | 9.71e-02 |
| A2 | FD+4 | FD+7 | 1.62 | 0.51 | [0.58, 2.66] | 3.45e-04 | 4.00e-04 | 1.62 | 0.51 | [0.59, 2.66] | 3.15e-04 | 4.00e-04 | 1.1 | 0.38 | [0.31, 1.88] | 2.15e-04 | 5.40e-03 |
| A2 | FD+7 | R+1 | -2.88 | 0.52 | [-3.95, -1.81] | 2.57e-06 | 0.00e+00 | -2.88 | 0.52 | [-3.95, -1.81] | 2.57e-06 | 0.00e+00 | 0.13 | 0.26 | [-0.41, 0.67] | 5.17e-01 | 6.32e-01 |
| A2 | R+1 | R+7 | -1.84 | 0.95 | [-3.79, 0.10] | 5.02e-02 | 6.07e-02 | -1.84 | 0.95 | [-3.79, 0.10] | 5.02e-02 | 6.08e-02 | -3.04 | 0.95 | [-4.99, -1.09] | 3.45e-04 | 3.10e-03 |
| A3 | L-45 | FD+4 | -1.66 | 0.34 | [-2.35, -0.96] | 8.66e-05 | 0.00e+00 | -1.64 | 0.34 | [-2.34, -0.94] | 8.66e-05 | 0.00e+00 | -1.66 | 0.37 | [-2.41, -0.91] | 1.60e-04 | 1.00e-04 |
| A3 | L-45 | FD+7 | -0.47 | 0.33 | [-1.14, 0.20] | 8.65e-02 | 1.68e-01 | -0.45 | 0.33 | [-1.12, 0.22] | 1.11e-01 | 1.88e-01 | -1.56 | 0.24 | [-2.05, -1.08] | 7.10e-07 | 0.00e+00 |
| A3 | L-45 | R+1 | -2.95 | 0.91 | [-4.81, -1.09] | 6.22e-03 | 2.90e-03 | -2.93 | 0.91 | [-4.80, -1.06] | 6.22e-03 | 3.50e-03 | -1.47 | 0.41 | [-2.31, -0.62] | 1.59e-03 | 2.00e-03 |
| A3 | L-45 | R+7 | -3.51 | 0.54 | [-4.63, -2.40] | 2.21e-06 | 0.00e+00 | -3.49 | 0.54 | [-4.60, -2.38] | 2.21e-06 | 0.00e+00 | -2.81 | 0.33 | [-3.48, -2.15] | 1.57e-07 | 0.00e+00 |
| A3 | FD+4 | FD+7 | 1.19 | 0.32 | [0.53, 1.84] | 4.14e-04 | 3.00e-04 | 1.19 | 0.32 | [0.54, 1.84] | 3.78e-04 | 3.00e-04 | 0.1 | 0.26 | [-0.44, 0.64] | 8.24e-01 | 7.17e-01 |
| A3 | FD+7 | R+1 | -2.48 | 0.72 | [-3.96, -1.01] | 2.50e-03 | 1.80e-03 | -2.48 | 0.72 | [-3.95, -1.01] | 2.70e-03 | 1.80e-03 | 0.1 | 0.2 | [-0.32, 0.52] | 9.61e-01 | 6.43e-01 |
| A3 | R+1 | R+7 | -0.56 | 1.14 | [-2.89, 1.76] | 8.85e-01 | 6.24e-01 | -0.56 | 1.14 | [-2.89, 1.76] | 8.85e-01 | 6.26e-01 | -1.35 | 0.32 | [-2.01, -0.69] | 3.57e-05 | 0.00e+00 |
| A4 | L-45 | FD+4 | 1.04 | 0.55 | [-0.09, 2.16] | 2.74e-02 | 7.24e-02 | 1.06 | 0.55 | [-0.07, 2.18] | 2.33e-02 | 6.67e-02 | 3.15 | 0.51 | [2.10, 4.20] | 6.52e-08 | 0.00e+00 |
| A4 | L-45 | FD+7 | 1.26 | 0.3 | [0.65, 1.87] | 7.78e-05 | 2.00e-04 | 1.28 | 0.3 | [0.68, 1.89] | 6.99e-05 | 0.00e+00 | 0.29 | 0.22 | [-0.16, 0.74] | 1.41e-01 | 2.08e-01 |
| A4 | L-45 | R+1 | -4.1 | 1.08 | [-6.30, -1.90] | 1.07e-03 | 4.00e-04 | -4.08 | 1.08 | [-6.27, -1.88] | 1.16e-03 | 4.00e-04 | -1.47 | 0.64 | [-2.78, -0.15] | 1.98e-02 | 2.59e-02 |
| A4 | L-45 | R+7 | 0 | 0.44 | [-0.91, 0.91] | 5.81e-01 | 1.00e+00 | 0.02 | 0.44 | [-0.88, 0.93] | 6.22e-01 | 9.59e-01 | 1.64 | 0.38 | [0.87, 2.41] | 1.97e-05 | 0.00e+00 |
| A4 | FD+4 | FD+7 | 0.23 | 0.43 | [-0.65, 1.11] | 5.42e-01 | 6.14e-01 | 0.23 | 0.43 | [-0.65, 1.11] | 5.42e-01 | 6.13e-01 | -2.86 | 0.38 | [-3.64, -2.08] | 9.31e-10 | 0.00e+00 |
| A4 | FD+7 | R+1 | -5.36 | 0.97 | [-7.34, -3.38] | 2.21e-06 | 0.00e+00 | -5.36 | 0.97 | [-7.34, -3.38] | 2.21e-06 | 0.00e+00 | -1.76 | 0.56 | [-2.91, -0.60] | 4.44e-03 | 2.70e-03 |
| A4 | R+1 | R+7 | 4.1 | 0.86 | [2.34, 5.86] | 1.05e-05 | 0.00e+00 | 4.1 | 0.86 | [2.34, 5.86] | 1.05e-05 | 0.00e+00 | 3.1 | 0.74 | [1.60, 4.61] | 3.57e-05 | 0.00e+00 |

Table S4. Variance explained DNA methylation-estimated immune cell types in the difference between epigenetic age acceleration and epigenetic intrinsic age acceleration.

| **Cell type** | **R-squared** |
| --- | --- |
| Treg | 0.207350237 |
| CD4Tnv | 0.164690496 |
| Neu | 0.108755784 |
| CD4Tmem | 0.086298132 |
| Mono | 0.079056595 |
| Bnv | 0.076292609 |
| CD8Tnv | 0.066746324 |
| Baso | 0.051135299 |
| CD8Tmem | 0.047479515 |
| Bmem | 0.045479435 |
| NK | 0.041027182 |
| Eos | 0.02369502 |

Supplementary Table 5. Mean change in age acceleration and intrinsic age acceleration between mission timepoints (Time 2 - Time 1) for each epigenetic clock category.

| **Category** | **Method** | **Time 1** | **Pre** | **Time 2** | **Post** | **Difference** |
| --- | --- | --- | --- | --- | --- | --- |
| Causal factors | Age acceleration | L-45 | -0.750133608 | FD+4 | -0.208581755 | 0.541551853 |
| Causal factors | Age acceleration | L-45 | -0.750133608 | FD+7 | 2.21652751 | 2.966661118 |
| Causal factors | Age acceleration | L-45 | -0.750133608 | R+1 | -0.14567497 | 0.604458638 |
| Causal factors | Age acceleration | L-45 | -0.750133608 | R+7 | -1.112137177 | -0.362003569 |
| Chronological age | Age acceleration | L-45 | -0.862523154 | FD+4 | 0.61007817 | 1.472601324 |
| Chronological age | Age acceleration | L-45 | -0.862523154 | FD+7 | 2.173979061 | 3.036502215 |
| Chronological age | Age acceleration | L-45 | -0.862523154 | R+1 | -0.974970838 | -0.112447685 |
| Chronological age | Age acceleration | L-45 | -0.862523154 | R+7 | -0.946563239 | -0.084040085 |
| Intrinsic age | Age acceleration | L-45 | 3.035164631 | FD+4 | 0.501561399 | -2.533603233 |
| Intrinsic age | Age acceleration | L-45 | 3.035164631 | FD+7 | 1.196089939 | -1.839074693 |
| Intrinsic age | Age acceleration | L-45 | 3.035164631 | R+1 | -2.128215374 | -5.163380005 |
| Intrinsic age | Age acceleration | L-45 | 3.035164631 | R+7 | -2.604600595 | -5.639765226 |
| Mortality | Age acceleration | L-45 | -0.716986607 | FD+4 | 0.606096944 | 1.323083551 |
| Mortality | Age acceleration | L-45 | -0.716986607 | FD+7 | 1.846334706 | 2.563321313 |
| Mortality | Age acceleration | L-45 | -0.716986607 | R+1 | -2.034119426 | -1.317132819 |
| Mortality | Age acceleration | L-45 | -0.716986607 | R+7 | 0.298674383 | 1.01566099 |
| Organ aging | Age acceleration | L-45 | 0.068278049 | FD+4 | 0.858633601 | 0.790355551 |
| Organ aging | Age acceleration | L-45 | 0.068278049 | FD+7 | 1.819909473 | 1.751631423 |
| Organ aging | Age acceleration | L-45 | 0.068278049 | R+1 | -2.988332619 | -3.056610669 |
| Organ aging | Age acceleration | L-45 | 0.068278049 | R+7 | 0.241511496 | 0.173233447 |
| Physical fitness | Age acceleration | L-45 | 1.624535584 | FD+4 | 0.174972384 | -1.4495632 |
| Physical fitness | Age acceleration | L-45 | 1.624535584 | FD+7 | 1.711180972 | 0.086645388 |
| Physical fitness | Age acceleration | L-45 | 1.624535584 | R+1 | -2.436086027 | -4.060621612 |
| Physical fitness | Age acceleration | L-45 | 1.624535584 | R+7 | -1.074602914 | -2.699138498 |
| Retroelements | Age acceleration | L-45 | 1.135385229 | FD+4 | -0.319598963 | -1.454984192 |
| Retroelements | Age acceleration | L-45 | 1.135385229 | FD+7 | 0.158724531 | -0.976660698 |
| Retroelements | Age acceleration | L-45 | 1.135385229 | R+1 | -1.438113796 | -2.573499025 |
| Retroelements | Age acceleration | L-45 | 1.135385229 | R+7 | 0.463602999 | -0.67178223 |
| Stochasticity | Age acceleration | L-45 | -1.169900041 | FD+4 | 1.139457496 | 2.309357537 |
| Stochasticity | Age acceleration | L-45 | -1.169900041 | FD+7 | 1.387885031 | 2.557785072 |
| Stochasticity | Age acceleration | L-45 | -1.169900041 | R+1 | 0.914197689 | 2.08409773 |
| Stochasticity | Age acceleration | L-45 | -1.169900041 | R+7 | -2.271640174 | -1.101740133 |
| Causal factors | Intrinsic age acceleration | L-45 | -0.885337118 | FD+4 | 1.495341423 | 2.380678541 |
| Causal factors | Intrinsic age acceleration | L-45 | -0.885337118 | FD+7 | 0.020175013 | 0.905512131 |
| Causal factors | Intrinsic age acceleration | L-45 | -0.885337118 | R+1 | 0.108212584 | 0.993549702 |
| Causal factors | Intrinsic age acceleration | L-45 | -0.885337118 | R+7 | -0.738391901 | 0.146945217 |
| Chronological age | Intrinsic age acceleration | L-45 | -0.142680855 | FD+4 | 0.976401644 | 1.119082499 |
| Chronological age | Intrinsic age acceleration | L-45 | -0.142680855 | FD+7 | 0.268818247 | 0.411499102 |
| Chronological age | Intrinsic age acceleration | L-45 | -0.142680855 | R+1 | -0.149760157 | -0.007079302 |
| Chronological age | Intrinsic age acceleration | L-45 | -0.142680855 | R+7 | -0.952778879 | -0.810098023 |
| Intrinsic age | Intrinsic age acceleration | L-45 | 0.902092569 | FD+4 | -0.048662716 | -0.950755285 |
| Intrinsic age | Intrinsic age acceleration | L-45 | 0.902092569 | FD+7 | 0.719596177 | -0.182496391 |
| Intrinsic age | Intrinsic age acceleration | L-45 | 0.902092569 | R+1 | -1.483411725 | -2.385504294 |
| Intrinsic age | Intrinsic age acceleration | L-45 | 0.902092569 | R+7 | -0.089614305 | -0.991706874 |
| Mortality | Intrinsic age acceleration | L-45 | 0.102324599 | FD+4 | 0.611614756 | 0.509290157 |
| Mortality | Intrinsic age acceleration | L-45 | 0.102324599 | FD+7 | -0.059876904 | -0.162201503 |
| Mortality | Intrinsic age acceleration | L-45 | 0.102324599 | R+1 | -0.194851346 | -0.297175944 |
| Mortality | Intrinsic age acceleration | L-45 | 0.102324599 | R+7 | -0.459211105 | -0.561535704 |
| Organ aging | Intrinsic age acceleration | L-45 | 0.110642534 | FD+4 | 0.372779899 | 0.262137366 |
| Organ aging | Intrinsic age acceleration | L-45 | 0.110642534 | FD+7 | 0.082628036 | -0.028014498 |
| Organ aging | Intrinsic age acceleration | L-45 | 0.110642534 | R+1 | -0.537457991 | -0.648100525 |
| Organ aging | Intrinsic age acceleration | L-45 | 0.110642534 | R+7 | -0.028592478 | -0.139235012 |
| Physical fitness | Intrinsic age acceleration | L-45 | 0.302269835 | FD+4 | 0.165497991 | -0.136771845 |
| Physical fitness | Intrinsic age acceleration | L-45 | 0.302269835 | FD+7 | 1.000887711 | 0.698617876 |
| Physical fitness | Intrinsic age acceleration | L-45 | 0.302269835 | R+1 | -1.681707266 | -1.983977101 |
| Physical fitness | Intrinsic age acceleration | L-45 | 0.302269835 | R+7 | 0.213051729 | -0.089218106 |
| Retroelements | Intrinsic age acceleration | L-45 | 0.638824804 | FD+4 | -0.404144584 | -1.042969388 |
| Retroelements | Intrinsic age acceleration | L-45 | 0.638824804 | FD+7 | 0.177292616 | -0.461532188 |
| Retroelements | Intrinsic age acceleration | L-45 | 0.638824804 | R+1 | -1.32568897 | -1.964513773 |
| Retroelements | Intrinsic age acceleration | L-45 | 0.638824804 | R+7 | 0.913716134 | 0.27489133 |
| Stochasticity | Intrinsic age acceleration | L-45 | -1.12255399 | FD+4 | 1.711354707 | 2.833908697 |
| Stochasticity | Intrinsic age acceleration | L-45 | -1.12255399 | FD+7 | 0.073128499 | 1.195682488 |
| Stochasticity | Intrinsic age acceleration | L-45 | -1.12255399 | R+1 | 0.355245083 | 1.477799073 |
| Stochasticity | Intrinsic age acceleration | L-45 | -1.12255399 | R+7 | -1.0171743 | 0.10537969 |

Figure S1. Distribution of epigenetic age estimates for each clock. Clocks are ordered by their correlation with chronological age (red labels).

Figure S2. Correlation between the biological age changes across astronauts and timepoints. Top panels display the pairwise Pearson’s correlations for EAD, EAA, IEAA. Bottom panels display the distribution of correlations stratified by comparison type (different individuals (any timepoint), different timepoints (any individual), same individual (any timepoint), and same timepoint (any individual)) for each measure (EAD, EAA, IEAA).

Figure S3. Changes in the pace of aging for each astronaut across timepoints. Points indicate individual DunedinPACE values by crew member.

Figure S4. DNA methylation estimated immune cell type proportions across timepoints. Each panel shows the predicted proportions for one of the 12 cell types.

Figure S5. Pairwise Pearson’s correlation between categories of epigenetic clocks for the biological age measures.

Figure S6. Average biological age change for different epigenetic clock categories.

Figure S7. Sensitivity analysis of biological age changes across epigenetic clocks using a leave-one-out approach.


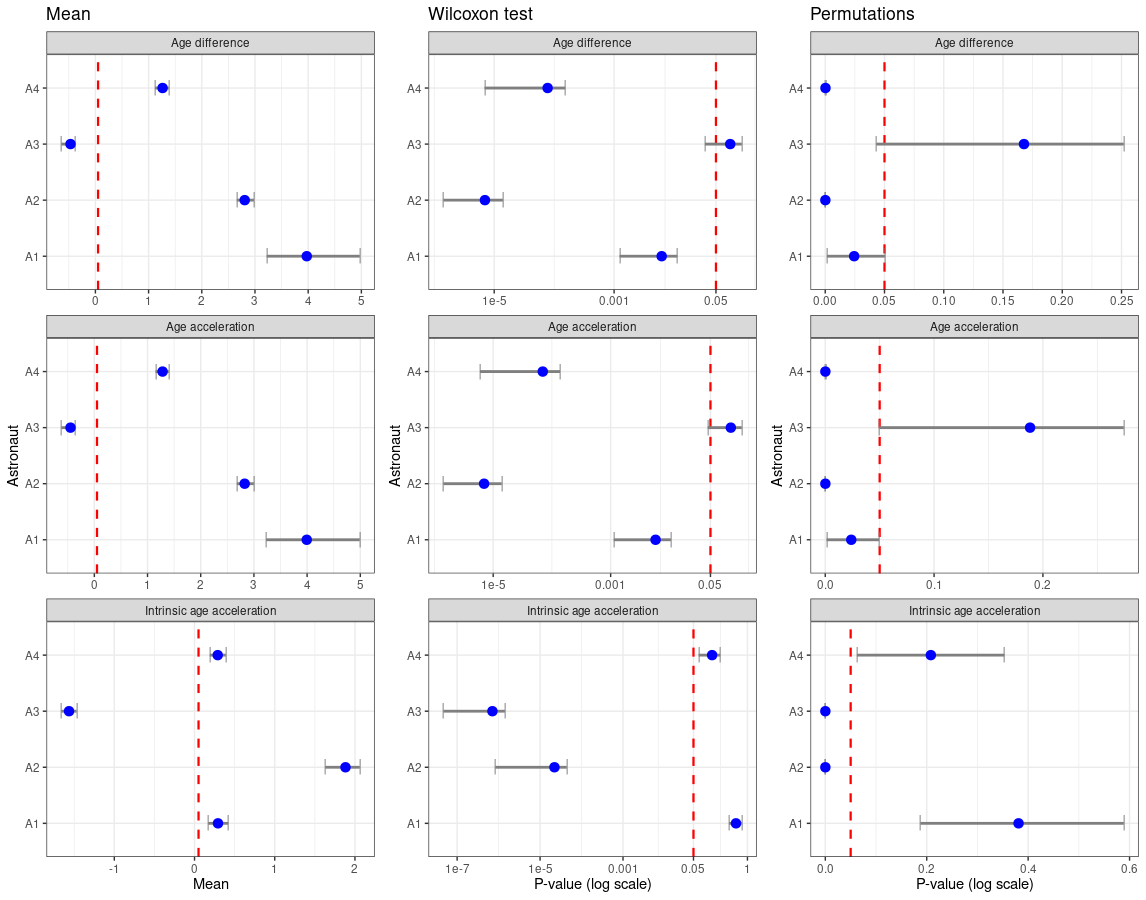

Supplement: Supplementary file 1 — Table S1: List of epigenetic clocks used in this study, with the corresponding original reference publication. Table S2: List of the 12 DNA methylation derived immune cell types used for cell composition adjustment. Table S3: Biological age differences for each crew member across different timepoints. For each biological age measure (EAD, EAA, and IEAA), the table describes the mean difference (Time 2—Time 1), standard error (SE), 95% confidence interval, and p values from paired Wilcoxon signed‐rank tests and sign‐flipping permutation tests on the paired differences in biological age. All p values reported in the main text correspond to the permutation‐based p values. Table S4: Variance explained DNA methylation‐estimated immune cell types in the difference between epigenetic age acceleration and epigenetic intrinsic age acceleration. Table S5: Mean change in age acceleration and intrinsic age acceleration between mission timepoints (Time 2—Time 1) for each epigenetic clock category. Figure S1: Distribution of epigenetic age estimates for each clock. Clocks are ordered by their correlation with chronological age (red labels). Figure S2: Correlation between the biological age changes across astronauts and timepoints. Top panels display the pairwise Pearson's correlations for EAD, EAA, and IEAA. Bottom panels display the distribution of correlations stratified by comparison type (different individuals (any timepoint), different timepoints (any individual), same individual (any timepoint), and same timepoint (any individual)) for each measure (EAD, EAA, and IEAA). Figure S3: Changes in the pace of aging for each astronaut across timepoints. Points indicate individual DunedinPACE values by crew member. Figure S4: DNA methylation estimated immune cell type proportions across timepoints. Each panel shows the predicted proportions for one of the 12 cell types. Figure S5: Pairwise Pearson's correlation between categories of epigenetic clocks for the biological age measures [file ACEL-25-e70360-s001.docx]
